# Heat acclimation prevents neurobehavioral and physiological disorders in mice exposed to chronic aircraft noise

**DOI:** 10.14814/phy2.70575

**Published:** 2025-10-02

**Authors:** Kangli Zhang, Xiaojing Lin, Ching‐Ping Chang, Xueqing Yi, Wei Wu, M. J. Walker, Qingjian Ding, Kai Liu, Chuanning Sun, Yanping Sun, Zhongya Shi, Cheng‐Hsien Lin, Gang Sun

**Affiliations:** ^1^ The Guidance Center for Military Psychology of PLA; Key Laboratory of Military Medical Psychology and Stress Biology of PLA; Department of Psychology, The 960th Hospital of Joint Logistics Support Force of PLA Jinan Shandong Province China; ^2^ Department of Medical Research Chi Mei Medical Center Tainan Taiwan; ^3^ Department of Medical Imaging The 960th Hospital of Joint Logistics Support Force of PLA Jinan Shandong Province China; ^4^ Department of Neurological Surgery, Spinal Cord and Brain Injury Research Group, Stark Neurosciences Research Institute Indiana University School of Medicine Indianapolis Indianan USA; ^5^ Big Data Analytics Laboratory of Sport Performance Assessment Sport College of Shandong University Jinan Shandong Province China; ^6^ Department of Applied Psychology, College of Sports and Health Shandong Sport University Jinan Shandong Province China; ^7^ Shandong Huayi Biotechnology Co., Ltd Jinan Shandong Province China; ^8^ Department of Medicine (Basic Sciences) Mackay Medical University New Taipei City Taiwan

**Keywords:** aircraft noise, blood–brain barrier disruption, endotoxemia, gut barrier disruption, heat acclimation, hippocampus, neurobehavioral disorders

## Abstract

We aimed to test whether heat acclimation (HA) would protect against aircraft noise (AN)‐induced neurobehavioral and physiological disorders in mice. A total of 90 adult male mice were equally divided into three groups: control (c) plus non‐AN group, c plus AN group, and HA plus AN group. Neurobehavioral performances included passive avoidance tasks (to assess learning and memory function), Y‐maze tests (to assess spatial memory ability), and novel object recognition tests. Physiological functions included stress responses, inflammation, and oxidative stress, which were determined biochemically. The severity of endotoxemia was determined by measuring the serum levels of lipopolysaccharide. Both gut barrier and blood–brain barrier permeability were determined by fluorescein isothiocyanate and Evans Blue dye measurement, respectively. Compared to c+non‐AN mice, the c+AN mice displayed neurobehavioral disorders along with exacerbated stress reactions, gut barrier disruption, endotoxemia, blood–brain barrier disruption, and hippocampal inflammation and oxidative stress. Compared to c+AN mice, the HA+AN mice had significantly less severity of all the abovementioned behavioral and physiological impairments. These results suggest that HA counteracts neurobehavioral and physiological disorders in mice exposed to aircraft noise.

## Graphical abstract

Effects of heat acclimation on the aircraft noise‐induced physiological and neurobehavioral disorders.

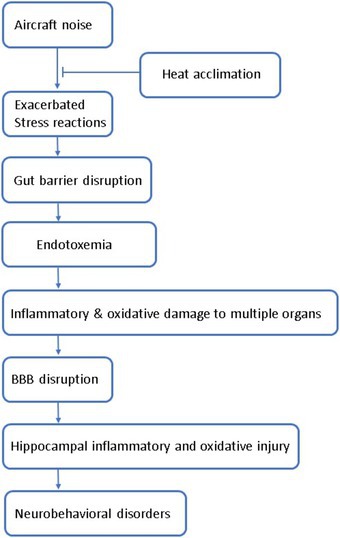

## 1 INTRODUCTION

Many previous investigations have shown that gut dysbiosis can cause reduced exploratory behavior, memory loss (Diaz Heijtz et al., 2011), anxiety‐like behavior (Hoban et al., 2016), and decreased locomotor activity (Ceylani et al., 2018). Rodents with chronic aircraft (AN) exposure also display cognitive deficits along with the exacerbated stress reactions, gut barrier disruption, endotoxemia (or increased serum levels of endotoxin), blood–brain barrier (BBB) disruption, and hippocampal inflammation and oxidative stress (Lin et al., 2022; Sun et al., 2021). Putting these observations together, it turns out that chronic AN exposure can cause cognitive deficits by affecting gut–brain axis functions.

Passive heat therapy has been described as capable of inducing heat acclimation (HA) and results in a reduction of physiological strain (Rodrigues et al., 2024). Heat acclimation has cross‐tolerance to subsequently applied stressors such as hypoxia or ischemic injury (Ely et al., 2014), closed head trauma (Shein et al., 2005), or myocardial damage (Mreisat et al., 2020). This raises the possibility that HA might counteract the chronic AN exposure‐induced physiological strain such as exacerbated stress reactions, gut barrier disruption, endotoxemia, BBB disruption, hippocampal inflammation, oxidative stress, and cognitive deficits.

In the present study, to deal with the hypothesis, heat acclimated mice, and non‐heat acclimated controls, were subjected to chronic AN exposure or non‐AN exposure. We investigated if HA prior to an AN exposure (8 h daily for 30 days) would protect mice against neurobehavioral and physiological disorders following an AN exposure.

## 2 | METHODS

### 2.1 Reagents

Cat numbers of EU‐TNF‐1 (Ray Biotech, USA), KE10003 (Proteintech, USA), and KE10007 (Proteintech, USA) assay kits were obtained for measurements of tumor necrosis factor (TNF‐α), interleukin‐1β (IL‐1β), and interleukin‐6 (IL‐6), respectively. Malonaldehyde (MDA) (#ab38176) assay kit, superoxide dismutase (SOD) activity assay kit (#ab65354), glutathione (GSH) assay kit (#ab13881), and 8‐hydroxy‐2‐deoxyquanoside (8‐OHdG) assay kit (#ab28285254) were purchased from Abcam (Cambridge, MA, USA). The total antioxidant capacity (T‐AOC) assay kit (#EEA022) was obtained from Thermo Fisher Scientific (Waltham, MA, USA). Evans blue dye (#E2129) was purchased from Sigma‐Aldrich (St Louis, MO, USA). All solvents and chemicals used in this study were of analytical grade.

### 2.2 Animals and housing

Male adult C57BL/6J mice (weighed 26–35 g, total number of 90) were obtained from Pengyue Biotechnology Co., Ltd., Jinan, Shandong, China, and kept at the animal facility on a 12 h: 12 h light/dark cycle. They were maintained at room temperature (24 ± 1°C), humidity (50 ± 5%), and room noise level (48 ± 1 dB). Behavioral tests were conducted after noise exposure—immediately afterward. All animals were treated following the Guide for the Care and Use of Laboratory Animals as adopted by the U.S. National Institutes of Health. Approval was granted by the Ethics Committee of the 960th Hospital of Joint Logistics Support Force of PLA, Shandong, China (permit No.: 2020‐10).

### 2.3 Heat acclimation

Heat acclimation was achieved by exposure to 34 ± 1°C and 30%–40% RH for 30 days (Horowitz, 1976; Mreisat et al., 2020). Normothermic or non‐HA control (C) groups of mice were maintained at 24 ± 1°C for 30 days. They were maintained in cages during heat acclimation on LabDiet 5010 mouse chow (Laboratory Autoclavable Rodent Diet 5010; Richmond, Indiana, USA).

### 2.4 Aircraft noise

The mice of the non‐AN group were exposed to homologous conditions without noise exposure (a mean sound pressure level the same as the animal house was approximately 48 dB). The mice in groups of two to three in their standard cages were moved to a testing room specified for chronic noise (a mean sound pressure level of 78 ± 1.0 dB) for 8 h daily at 8:00 am–16:00 pm for 30 days (Steven et al., 2020; Zhang et al., 2025). Noise was applied through downward‐facing speakers positioned approximately 30 cm above open mouse cages. The mice in the non‐AN group were left undisturbed for 8 h starting at 8:00 am to 16:00 pm for 30 days. Food and water were provided during the testing period.

### 2.5 Experimental groups and procedures

A total of 90 mice were assigned to three groups and designed as follows: control (C) 24^o^C + non‐AN group (C+non‐AN), control 24^o^C + AN group (C+AN), and heat acclimation (HA) 34^o^C + AN group (HA+AN).

**FIGURE 1:**
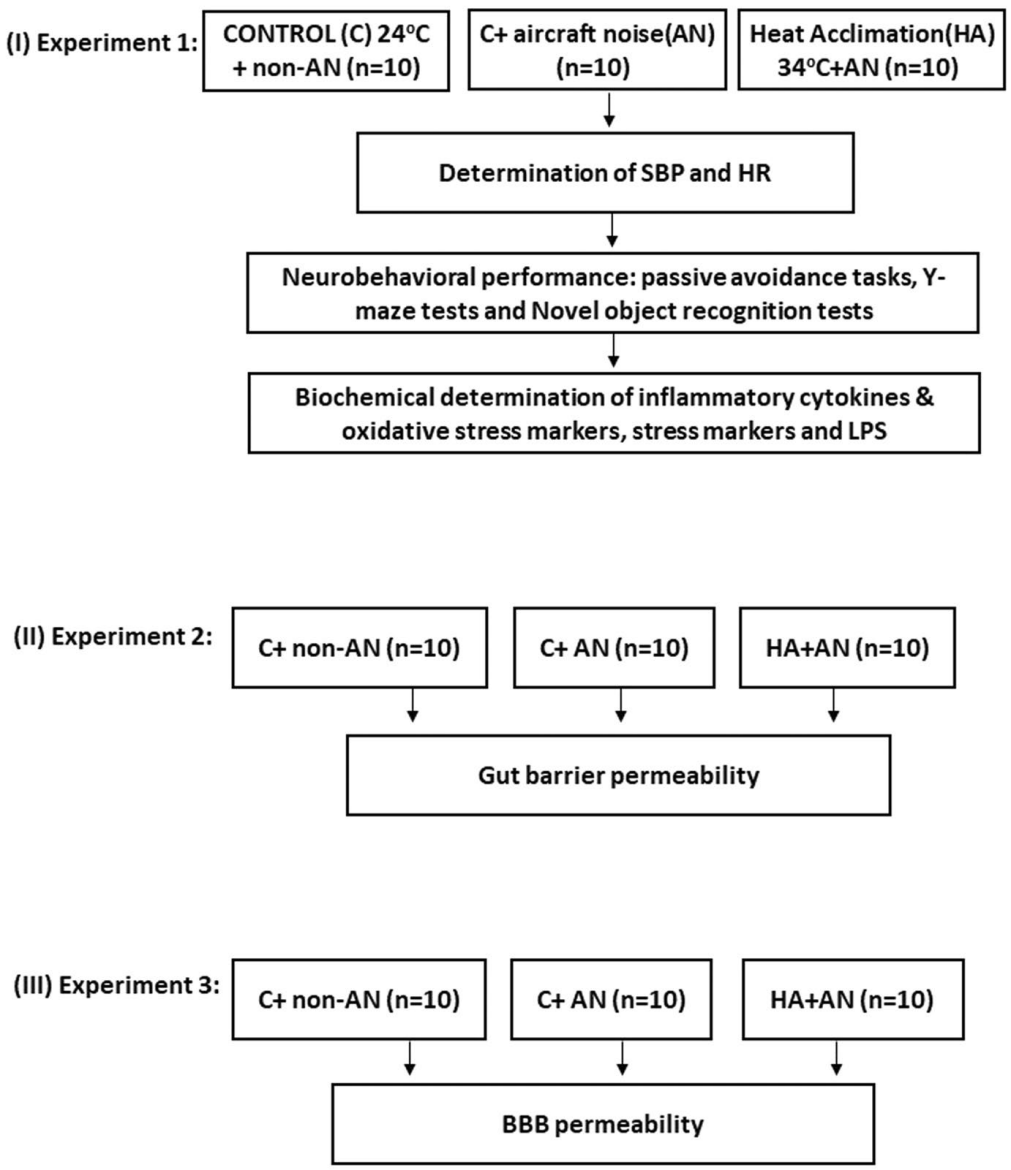
Experimental scheme. Mice undergoing HA or C with AN or without AN (non‐AN) were assigned to three different experimental paradigms. In experiment 1, 1 day following the last aircraft noise (AN) exposure, we first determined the values of both systolic blood pressure (SBP) and heat rate (HR) in C + non‐AN (*n* = 8), C + AN (*n* = 10), and HA + AN (*n* = 10) groups of mice. Then, these groups of mice were subjected to neurobehavioral tests. One day following the neurobehavioral trials, these groups of mice were euthanized and their blood and tissue samples were collected for determination of values of pro‐inflammatory cytokines, oxidative damage markers, stress indicators, and lipopolysaccharide (LPS). In our experiment 2 and experiment 3, the measurements of gut barrier permeability and blood–brain barrier (BBB) permeability were determined respectively in the C+non‐AN group (*n* = 10 each), C+AN group (*n* = 10 each), and HA+AN group of mice (*n* = 10 each).

As depicted in Figure 1, in our experiment 1, we determined the values of systolic blood pressure (SBP) and heart rate (HR) from the C+non‐AN (*n* = 10), C+AN (*n* = 10), and HA+AN (*n* = 10) groups of mice at 1 day after the last noise exposure. Immediately after the determination of physiological values. We performed a behavioral test battery in the following order: passive avoidance tasks, Y‐maze tests, and novel object recognition tests. One day following the neurobehavioral performance, the mice of these three groups were euthanized via an overdose of Zoletil (20 mg/kg body weight, i.p.). Blood and tissue samples were collected for determination of values of pro‐inflammatory cytokines and oxidative stress markers. In our experiment 2, the gut barrier permeability of the C+non‐AN (*n* = 10), C+AN (*n* = 10), and HA+AN group of mice (*n* = 10) groups of mice were determined at 1 day following the noise exposure. In our experiment 3, 1 day following noise exposure, the mice of the C+non‐AN (*n* = 10), C+AN (*n* = 10), and HA+AN (*n* = 10) were subjected to blood–brain barrier permeability measurement.

### 2.6 Behavioral tests

Several neurobehavioral functions including learning and memory ability (by passive avoidance tests), spatial memory ability (by Y‐maze tests), and novel object recognition tests were assessed.

Learning and memory functions were assessed by a two‐compartment step‐through passive avoidance apparatus. During the acquisition time assessment, when the hind leg of the mice entered the dark, the guillotine door was closed and an electric foot shock was delivered. Twenty‐four hours following the acquisition time assessment, the mice were tested in the bright chamber for the retention trial as described previously (Hosseini et al., 2013). The Y‐maze tests were performed to assess spatial memory function. The latency to enter the correct arm (or time of reaction) and the number of wrong entries (or error numbers) were determined by a three‐arms arranged Y‐maze (Wei et al., 2012). For assessment of the exploration time of the novel object and the novel recognition index (Choi et al., 2011), animals were put into the test box with two identical objects for 3 min. One day after the session, animals were allowed to explore a familiar object and a novel object for 3 min. The time that the animal spent exploring the familiar (t familiar) and the novel object (t novel) was measured.

### 2.7 Stress reactions

Animals were habituated in the restrainer and familiarized with the tail‐cuff (NIBP system with AD Instruments) for measuring systolic blood pressure (SBP) and heart rate (HR) (Chauhan et al., 2017). Blood samples were collected from the abdominal aorta of previously anesthetized mice with Zoletil® 100 (10 mg/kg; Virbac, Carros, France) in plain tubes. The serum was used to assess adrenocorticotropic hormone (ACTH), corticosterone, and norepinephrine using a mouse ACTH ELISA kit (EEL085; Jishan Science and Technology, Bejing, China), a mouse corticosterone Rapid ELISA kit (EELR008; Jishan Science and Technology), and a norepinephrine ELISA kit (ab287789; Abcam, Cambridge, MA, USA), respectively. A hemolysis‐free serum was generated by centrifugation of blood using serum separator tubes (Becton Dicherson, Franklin Lakes, NJ, USA). On the day of analysis, serum was diluted in lipopolysaccharide (LPS)‐free saline. The serum levels of LPS were determined by a mouse LPS ELISA kit (CSB‐E 13066m, Jinshan Science and Technology Co., Ltd., Beijing, China) according to the manufacturer's instructions (Caricilli et al., 2011).

### 2.8 Pro‐inflammatory cytokines and oxidative stress indicators

The heart, duodenum, and hippocampus tissues of the mice under general anesthesia (Zoletil 20 mg/kg, i.p.) were isolated and stored at −80°C until use. The cardiac, intestinal, and hippocampal levels of tumor necrosis factor‐α (TNF‐α; #EU‐TNF‐1, Ray Biotech, USA), interleukin‐1β (IL‐1β; #KE10003; Proteintech, USA), interleukin‐6 (IL‐6; #KE10007, Proteintech, USA), malonaldehyde (MDA, #ab38176, Abcam, Cambridge, MA, USA), superoxide dismutase (SOD; #ab65354, Abcam, Cambridge, MA, USA), glutathione (GSH, #ab13881, Abcam, Cambridge, MA, USA), 8‐hydroxy‐2‐deoxyguanosine (8‐OHdG; #ab28285254, Abcam, Cambridge, MA, USA), and total antioxidant capacity (T‐Aoc; #EEA022; Abcam, Cambridge, MA, USA) were quantified using enzyme‐linked immunosorbent assay (ELISA) kits according to the manufacturer's instructions.

### 2.9 Blood–brain barrier (BBB) and intestinal permeability

For assessments of BBB permeability, at first, Evans Blue dye (2%) (#E2129, Sigma‐Aldrich Co., St. Louis, MO, USA) was injected into the tail vein and allowed to circulate for 1 h in a mouse under general anesthesia (Zoletil 10 mg/kg, i.p.; #304801) (Virbac, Nice, France). Then, the dye was removed from the circulation by intracardiac perfusion of saline. The brain was removed and incubated in formamide (Sigma) in a water bath. A spectrophotometer was used to measure the Evans Blue content in the supernatants (Goldim et al., 2019). It was presented as μg of Evans Blue dye per g of tissue.

For assessments of intestinal permeability, mice were killed by an intraperitoneal dose of Zoletil (20 mg/kg). The segments of the duodenum were obtained. The mucosa was everted from each and ligated at one end, and from the other end, a gut sac was prepared by injection of buffer solution. The filled sac was incubated in a solution containing fluorescein‐isothiocyanate (FD‐4) (#E3658, Sigma‐Aldrich, MO, USA). The mucosa‐to‐serosal clearance of FD‐4 in terms of nl/min/cm^2^ was used to determine intestinal permeability (Tan et al., 2016).

### 2.10 Statistical analysis

Blinding was used to collect and analyze the data for all behavioral and biochemical protocols. Results are expressed as the mean ± SD Behavioral data are tested for normality (D'Agostino & Pearson Omnibus normality test) and skewness using GraphPad 7.01 (GraphPad Software, San Diego, USA). We used a 1‐way ANOVA plus Tukey's multiple comparisons tests to compare the data, with *p* < 0.05 considered statistically significant.

## 3 RESULTS

### 3.1 Neurobehavioral functions

**FIGURE 2:**
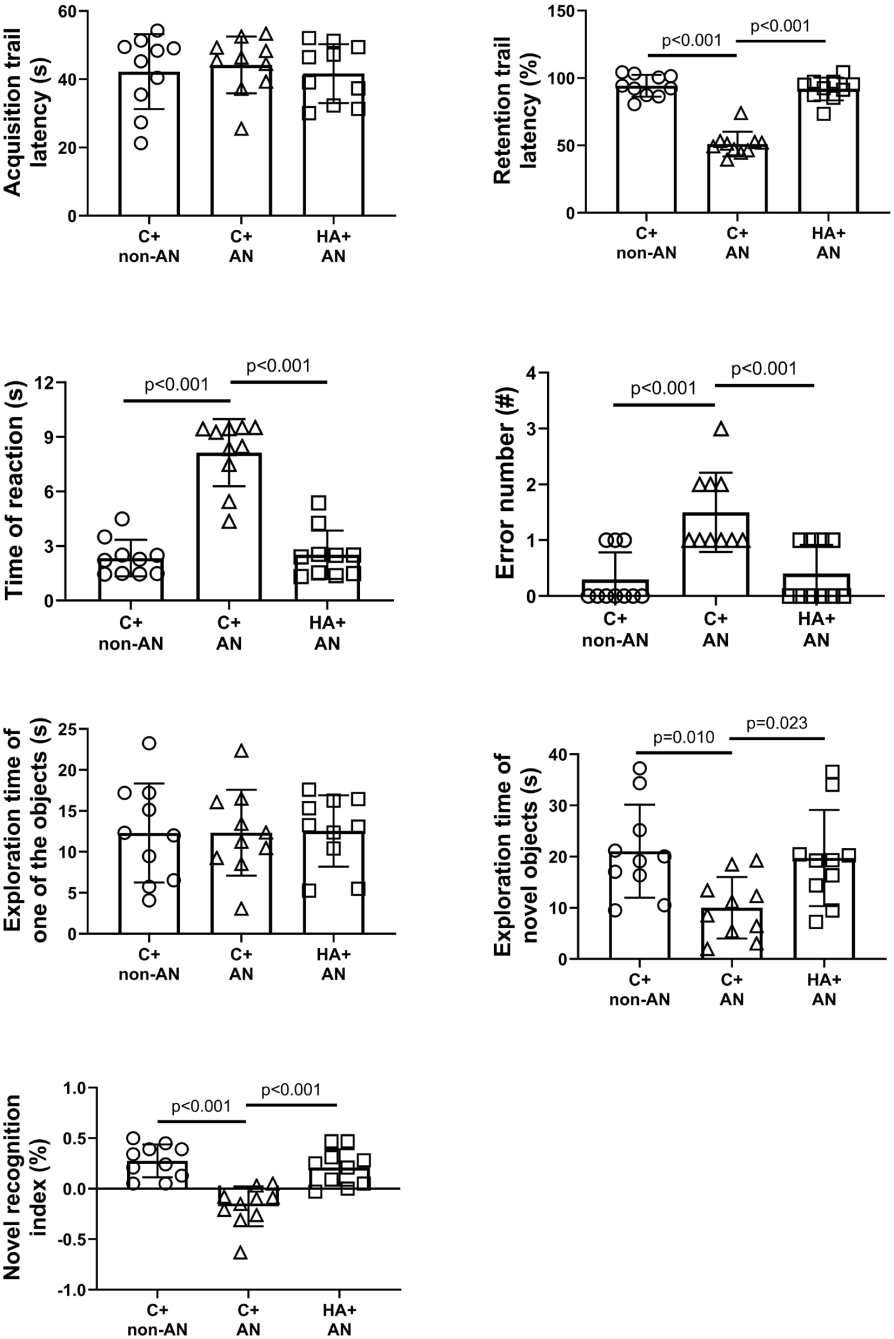
Effects of AN on neurobehavioral functions in mice. Mice were subjected to a battery of neurobehavioral performances (passive avoidance task, Y‐maze tests, and NOR tests). Values are presented as mean ± SD of *n* = 10 for each group.

We determined the latency to entering the dark chamber before receiving the foot shock (preshock; acquisition time; Figure 2) and 24 h after receiving the foot shock (after 24 h retention time; Figure 2) by a passive avoidance task. The C+AN group of mice had a significantly lower retention trial latency (Figure 2). Compared to the C+AN group of mice, the HA+AN group of mice had a significantly higher retention trial latency (Figure 2).

The Y‐maze tests showed that the C+AN group of mice had significantly higher values of both time of reaction (Figure 2) and error numbers compared with the C+non‐AN group mice (Figure 2). Compared to the C+AN group of mice, the HA+AN group of mice had significantly lower values of both time of reaction and error numbers (Figure 2).

The NOR tests showed that the C+AN group of mice had significantly lower values of both the exploration time of novel objects and the novel recognition index than did the C+non‐AN group of mice (Figure 2). However, compared to the C+AN group of mice, the HA+AN group of mice had significantly higher values of both the exploration time of novel objects and the novel recognition index (Figure 2).

### 3.2 Stress reactions

**FIGURE 3:**
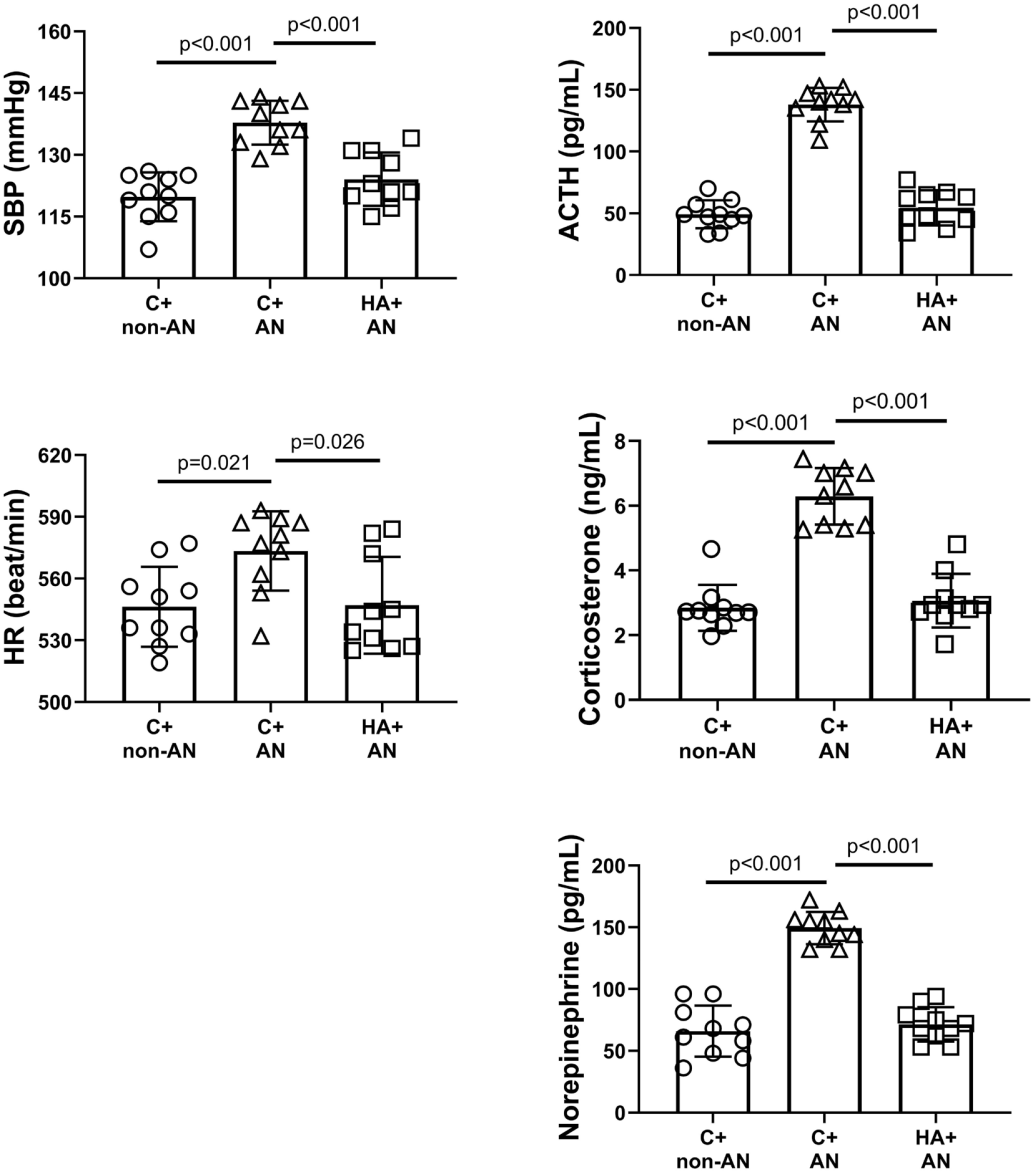
Systolic blood pressure (SBP), heart rate (HR), plasma ACTH, plasma corticosterone, and plasma noradrenaline values in C+non‐AN mice, C+AN mice, and HA+AN mice. The values were obtained 7 days after initiation of AN. Bars are the mean ± SD of *n* = 10 for each group.

We determined stress reactions including SBP, HR, and plasma values of ACTH, corticosterone, and norepinephrine for different groups of mice. Compared to the C+non‐AN group of mice, the C+AN group of mice had significantly higher values of SBP, HR, and plasma levels of ACTH, corticosterone, and norepinephrine (Figure 3). However, the HA+AN group of mice had significantly lower values of all these stress reaction parameters than did the C+AN group of mice (Figure 3).

### 3.3 Tissue levels of pro‐inflammatory cytokines

**FIGURE 4:**
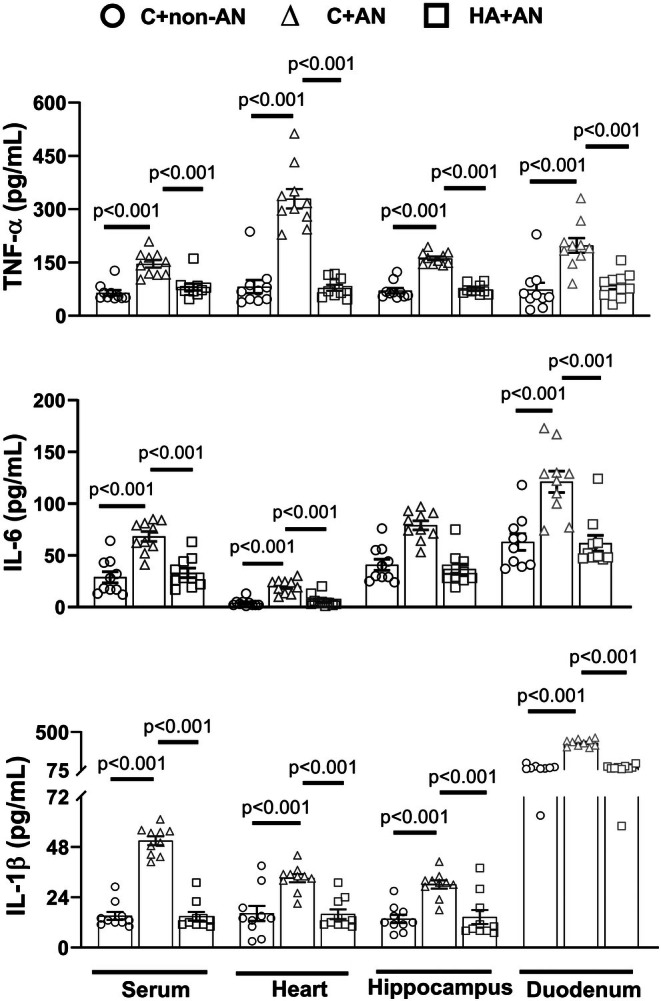
Effects of aircraft noise (AN) on the levels of IL‐6, TNF‐α, and IL‐1β in the heart, duodenum and hippocampus in C+non‐AN mice, C+AN, and HA+AN. The values were obtained 7 days after the iniation of AN. Bars are the mean ± SD of *n* = 10 for each group.

We determined the levels of pro‐inflammatory cytokines (e.g., TNF‐α, IL‐6, and IL‐1β) in the serum, heart, hippocampus, and duodenum of different groups of mice. Compared to the C+non‐AN group of mice, the C+AN group of mice had significantly higher values of all pro‐inflammatory cytokines (Figure 4). However, the HA+AN group of mice had significantly lower values of all tissue pro‐inflammatory cytokines (Figure 4).

### 3.4 Tissue levels of the oxidative damage indicators and antioxidant indicators in mice under an AN exposure

**FIGURE 5:**
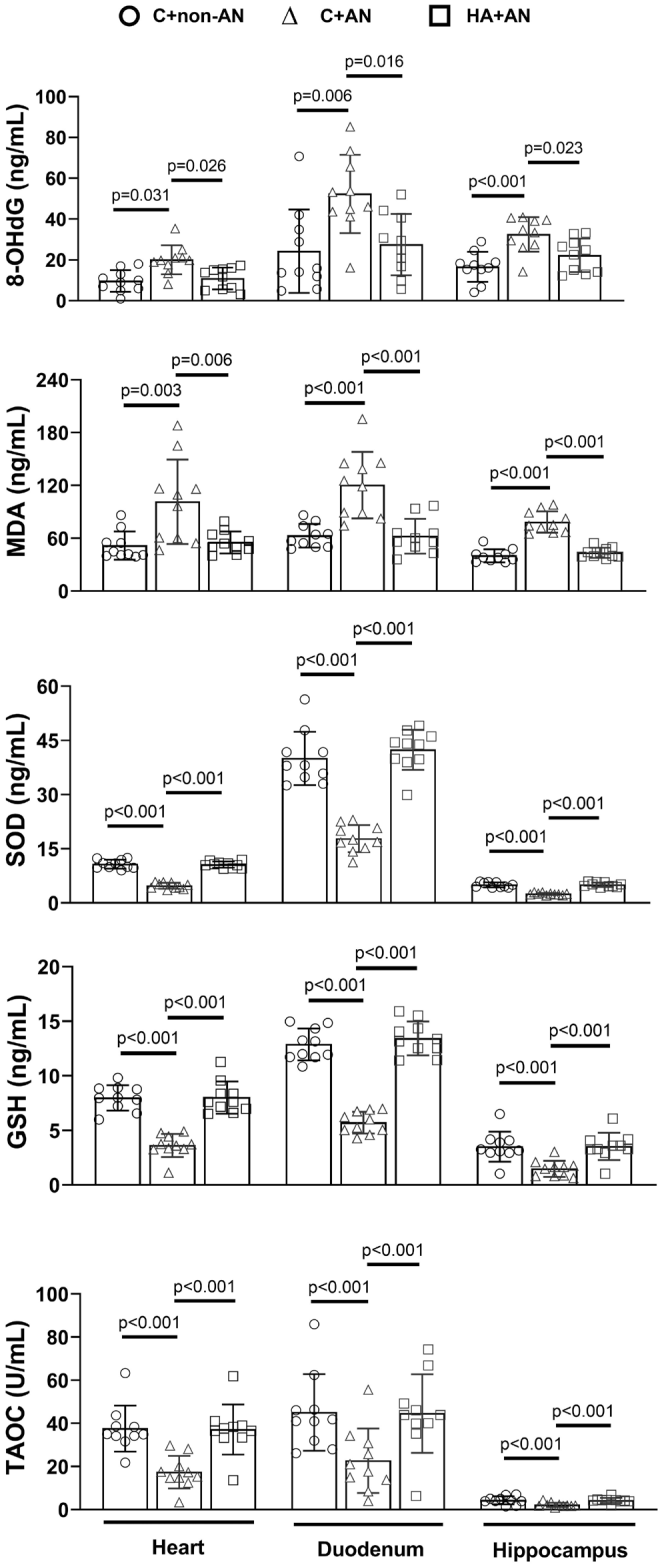
Effects of AN on the levels of 8‐OHdG, MDA, H_2_O_2_, SOD, GSH, and T‐AOC in the mouse heart, duodenum, and hippocampus in C+non‐AN mice, C+AN mice, and HA+AN mice. The values were obtained 7 days after the initiation of AN. Bars are the mean ± SD of *n* = 10 for each group.

We showed that the levels of the oxidative damage indicators (e.g., 8‐OHdG and MDA) and antioxidant indicators (e.g., SOD, GSH, and T‐AOC) in the heart, duodenum, and hippocampus of the C+AN group of mice were significantly higher and lower, respectively, than those of the C+non‐AN group of mice (Figure 5). However, compared to the C+AN group of mice, the HA+AN group of mice had significantly lower values of oxidative damage indicators but significantly higher values of anti‐oxidant indicators (Figure 5).

### 3.5 Gut barrier permeability, blood–brain barrier permeability, and plasma levels of endotoxin

**FIGURE 6:**
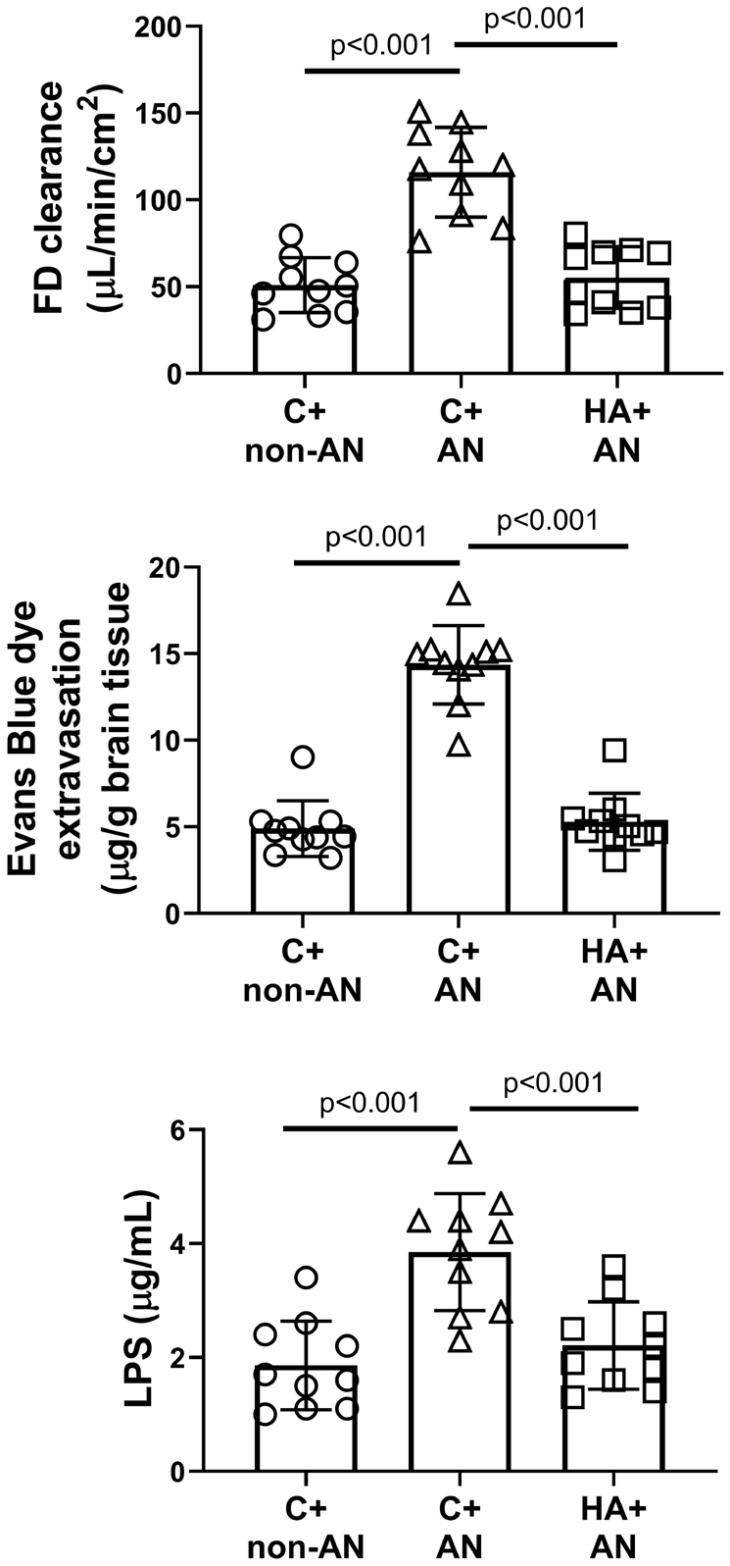
Effect of AN on the gut barrier permeability, the blood–brain barrier permeability, and plasma levels of LPS in C+non‐AN mice, C+AN mice, and HA+AN mice. Values are expressed as mean ± SD of *n* = 10 per group.

We determined the gut barrier permeability, BBB permeability, and plasma level of endotoxin by measuring intestinal FD clearance, cerebral Evans Blue dye extravasation, and plasma lipopolysaccharide (LPS), respectively. Compared to the C+non‐AN, the C+AN group of mice had significantly higher values of intestinal FD clearance, cerebral Evans Blue dye extravasation, and increased plasma levels of LPS (Figure 6). However, the HA+AN group of mice had significantly lower values of these abovementioned parameters than did the C+AN group of mice (Figure 6).

## 4 DISCUSSION

Previous results demonstrated that HA caused a reduction in physiological strain (Rodrigues et al., 2024), hypoxia or ischemic injury (Ely et al., 2014), closed head trauma (Shein et al., 2005; Umschweif et al., 2014), or myocardial injury (Mreisat et al., 2020). Our present results further showed that HA reduced chronic AN‐induced learning and memory dysfunction (evaluated by passive avoidance tasks), spatial memory loss (evaluated by Y‐maze tests), and recognition impairments (evaluated by novel object recognition tests), along with the exacerbated stress reactions, gut barrier disruption, endotoxemia, BBB disruption, and hippocampal neuroinflammation and oxidative stress.

Whole brain radiotherapy can cause significant cognitive deficits in up to 90% of patients who undergo the treatment (Perry & Schmidt, 2006; Smart, 2017). In rats, brain radiation can also cause cognitive deficits (Ho et al., 2025). These cognitive impairments are likely linked to hippocampal injury caused by irradiation. On the other hand, precise irradiation of the 22 × 10 cm field's center of interest to the pelvic region in rats can also cause alterations in microbial diversity, disrupted intestinal morphology and integrity, neuronal death‐related brain changes, neuroinflammation, and reduced locomotor activity (Venkidesh et al., 2023). Other environmental noise or stressors further displayed deteriorative effects on multiple organ functions, including healing (Fritschi et al., 2011), cardiovascular and neurobehavioral function (Babisch, 2014; Diaz‐Moran et al., 2014; Farazi et al., 2022; Jafari et al., 2018; Mahmoodzadeh et al., 2021). Several other stressors also caused HPA activation (Ait‐Belgnaoui et al., 2014), exacerbated stress reactions (Kelly et al., 2015; Vanuytsel et al., 2014), increased plasma, corticosterone, and endotoxemia (Moussaoui et al., 2014), and brain neuroinflammation (Kelly et al., 2015; Vanuytsel et al., 2014). Our present results further demonstrate that chronic AN exposure can cause exacerbated stress reactions, gut barrier disruption, endotoxemia, BBB disruption, hippocampal inflammation and oxidative stress, and neurobehavioral disorders in mice. Putting these observations together, environmental noise or stressors can cause neurobehavioral disorders along with physiological disorders such as exacerbated stress reactions, intestinal dysmorphology, gut barrier disruption, BBB disruption, endotoxemia, and brain damage. All of the abovementioned physiological and neurobehavioral impairments caused by noise exposure or other stressors might be counteracted by HA.

Both hippocampal degeneration and neurobehavioral disorders were noted in psychological patients following trauma (Gilbertson et al., 2002) or rodents following restraint (Conrad et al., 1999) or chronic traffic noise (Farazi et al., 2022; Jafari et al., 2018; Mahmoodzadeh et al., 2021). Herein, our present results further showed that chronic AN exposure greatly elevated inflammatory cytokines (such as IL‐1β, IL‐6, and TNF‐α) and oxidative damage indicators (e.g., 8‐OHdG and MDA) but reduced both antioxidant enzymes (e.g., SOD and GSH) and antioxidant activity indicators (e.g., T‐AOC) in the mouse hippocampus. In addition, HA counteracted the AN‐induced neurobehavioral disorders in mice via attenuating hippocampal neuroinflammation and oxidative stress (Figures 4, 5).

Patients with hypertension have been found to have higher plasma levels of norepinephrine (Makino et al., 2006). Additionally, the incidence of heart diseases and hypertension is directly related to AN exposure (Franssen et al., 2004; Knipschild, 1977). Chronic noise exposure can induce decreased behavioral activity, increased anxiety levels, and spatial memory loss in rats (Manukyan et al., 2020). Anxiety‐induced plasma norepinephrine augmentation increases radical oxygen species formation by monocytes in hypertension (Yasunari et al., 2006). In rodents under chronic noise exposure, administration of α‐adrenoreceptor blockers attenuated the elevated anxiety and restored impaired spatial memory (Manukyan et al., 2020). Our present results demonstrated that HA inhibited the AN‐induced increased plasma norepinephrine, hypertension, and neurobehavioral disorders in mice.

Heat acclimation mediated cardioprotection is controlled by mitochondrial metabolic remodeling involving hypoxia inducible factor (HIF‐1α) (Mreisat et al., 2020) and heat shock protein‐70 (Huang et al., 2025). The molecular mechanisms underlying neuroprotection include reduced inflammation and apoptosis (Horowitz, 2014; Umscheif et al., 2010), induction of HIF‐1α, brain‐derived neurotrophic factor (BDNF), Akt phosphorylation, erythropoietin signaling, and angiotensin receptor type 2 signaling (Umschweif et al., 2014). Our present results further show that HA might inhibit chronic AN‐induced cognitive deficits via affecting tissue levels of norepinephrine, ACTH, corticosterone (exacerbated stress reactions), gut barrier permeability, LPS (endotoxemia), pro‐inflammatory cytokines, and oxidative stress indicators. It is likely that the molecular mechanisms underlying HA‐mediated cognitive protection might involve HIF‐α, BDNF, Akt phosphorylation, erythropoietin signaling, and angiotensin receptor type 2 signaling. Recently, it has been promoted that passive heat therapy can be a promising preventive measure for people at risk of adverse health outcomes during heat extremes (Rodrigues et al., 2024). Herein we further demonstrate that HA (passive heat related) can act as a preventive measure for mice at risk of neurobehavioral and physiological disorders during a chronic noise exposure.

In humans, physiological adaptation associated with HA includes a lower resting core temperature, a greater sweating capacity, and a reduced heart rate during heat exposure (Rodrigues et al., 2024). These adaptations could minimize the health risks associated with extreme heat. In this context, in mice, HA offers a preventive measure at risk of cognitive deficits during a chronic AN exposure. Some translational limitations include the fact that the mouse exhibits distinctive thermoregulatory characteristics (e.g., variable core temperature, high metabolic rate, and thermal conductance) and attenuated inflammatory responses during a second heat illness if retested within 7 days (Caldwell et al., 2021). However, studies indicate that patients with heat illness present acute neurological impairments such as motor or cognitive deficits (Lawton et al., 2019). Research in this domain still needs further investigation.

There may be sex differences in the beneficial effects of HA protection in chronic AN exposure. For example, while an increased enteric load of LPS increases anxiety‐like behavior in mice of both sexes, it does so via sex‐specific mechanisms (Fields et al., 2018). Therefore, future studies should test HA triggers physiological and behavioral changes in males as well as females, but the underlying signaling mechanisms may differ.

In summary, our findings showed that chronic noise exposure caused exacerbated stress reactions, gut barrier disruption, endotoxemia, BBB disruption, and inflammatory and oxidative injury to many vital organs, which can act as causative factors for the occurrence of neurobehavioral disorders such as learning and memory dysfunction, spatial memory loss, and impaired novel object recognition ability. Heat acclimation not only significantly attenuated chronic noise exposure‐induced neurobehavioral disorders but also inhibited AN‐induced exacerbated stress reactions, gut barrier disruption, endotoxemia, BBB disruption, and hippocampal inflammation and oxidative stress. This indicates that HA can be a promising preventive method for people at risk of adverse health outcomes during noise exposure.

## AUTHOR CONTRIBUTIONS

Kangli Zhang, Xiaojing Lin, Ching‐Ping Chang, Cheng‐Hsien Lin, and Gang Sun were involved in conception, funding acquisition, supervision, and writing review editing. Xueqing Yi, Wei Wu, M.J. Walker, Qingjian Ding, Kai Liu, Chuanning Sun, Yanping Sun, and Zhongya Shi performed experiments, analyzed data, and prepared figures. Cheng‐Hsien Lin, Xiaojing Lin, Cheng‐Hsien Lin, and Gang Sun interpreted results of experiments and drafted manuscript. Ching‐Ping Chang, Xiaojing Lin, Kangli Zhang, Cheng‐Hsien Lin, and Gang Sun edited and revised manuscript. Ching‐Ping Chang, Cheng‐Hsien Lin, and Gang Sun approved final version of manuscript and correspondence.

## CONFLICT OF INTEREST STATEMENT

The authors declare no competing interests.

## ETHICS STATEMENT

All protocols involving the use of animals compiled with the Guide for the Care and Use of Laboratory Animals published by the National Institutes of Health (NIH) (DHEW publication NIH 85‐23‐2985) and were approved by the 960th Hospital of Joint Logistics Support Force of PLA, Shandong, China Animal Use Committee.

## Data Availability

Data will be available upon request.
